# A Colorimetric Membrane-Based Sensor with Improved Selectivity towards Amphetamine

**DOI:** 10.3390/molecules26216713

**Published:** 2021-11-05

**Authors:** Neus Jornet-Martínez, Pilar Campíns-Falcó, Rosa Herráez-Hernández

**Affiliations:** MINTOTA Research Group, Departament de Química Analítica, Universitat de València, Dr. Moliner 50, 46100 Burjassot, Spain; Neus.Jornet@uv.es (N.J.-M.); pilar.campins@uv.es (P.C.-F.)

**Keywords:** amphetamine, colorimetric sensors, gold bromide, drug analysis, illicit drug samples

## Abstract

Due to their simplicity, speed and low cost, chemical spot tests are increasingly demanded for the presumptive identification of illicit drugs in a variety of contexts such as point-of-care assistance or prosecution of drug trafficking. However, most of the colorimetric reactions used in these tests are, at best, drug class selective. Therefore, the development of tests based on chemical reactions with improved discrimination power is of great interest. In this work, we propose a new colorimetric assay for amphetamine (AMP) based on its reaction with solutions of alkaline gold bromide to form an insoluble yellow–orange derivative. The resulting suspensions are then filtered onto nylon membranes and the precipitate collected is used for the visual identification of AMP. The measurement of the absorbance of the membranes by diffuse reflectance spectroscopy also allows the quantification of AMP in a simple and rapid way, as demonstrated for different synthetic and drug street samples. On the basis of the results obtained, it was concluded that the proposed procedure is highly selective towards AMP, as this compound could be easily differentiated from other common drugs such as methamphetamine (MET), ephedrine (EPH), scopolamine (SCP) and cocaine (COC).

## 1. Introduction

The consumption of illicit drugs, and thus, the criminal activities associated with their production and marketing, continue to grow each year [1]. As a result, there is an increasing demand for analytical methodologies applicable to a variety of contexts such as the investigation of consumption habits, the monitoring of rehabilitation treatments or the characterization of seizures, among others [1,2]. The analytical strategy to be applied depends on the intended purpose of the analysis, and a variety of approaches are in current use, from those involving highly sophisticated chromatographic equipment coupled to spectrometers and/or chemometric data treatment [3,4,5] to simple chemical spot tests [6,7]. The former approaches are typically used to analyze complex matrices such as biological specimens or for confirmatory purposes, whereas because of their simplicity, speed and low cost, chemical spot tests are preferably used for the presumptive analysis of suspected samples. Chemical tests provide valuable information in a variety of situations (quite often in the field) such as medical emergencies, the rapid characterization of seizures, and on the roadside or workplace tests. They are also used to reduce the workload of many laboratories by avoiding negative samples to be analyzed by instrumental methods [8,9].

In recent years, detection strategies based on colorimetric reactions have been undergoing rapid development in parallel to the introduction of new analytical tools, mainly portable miniaturized instruments and sensor devices [10]. As a result, several methods were described for the analysis of drugs based on microfluidic devices [11,12] and solid sensors [13,14,15,16,17,18,19], often combined with digital imaging testing technology.

Despite their popularity, colorimetric methods present some drawbacks such as the risks associated with the manipulation of harmful reagents and their low discrimination power. The reactions involved in the generation of the response with many of the most popular tests are, at best, drug class selective. An example is the Marquis test, which is considered the colorimetric test most frequently used in the analysis of unknown samples [6]. This test is based on the colors observed when the sample is treated with solutions of formaldehyde and sulfuric acid and allows for the identification of opiates and amphetamines. However, drugs with similar structures produce derivatives with similar colors. For example, AMP and MET form orange-brown products, and thus they cannot be differentiated with this test [12,18]. The Marquis reagent is also reactive towards some additives and adulterants.

In order to overcome the lack of selectivity of colorimetric assays, different approaches were proposed. The development of new recognition probes such as aptamers has attracted a lot of attention [6,7]. Extensive research was also carried out to develop new (nano)materials and sensor devices with enhanced detection capabilities [20]. From another perspective, taking advantage of the simplicity and low cost of colorimetric assays, different devices were developed capable of combining two or more recognition reactions in order to generate a multi-response, thus providing enhanced discrimination power. For example, a paper analytical device that can run six tests simultaneously was developed by Musile et al. for the identification of some common drugs [11]. The quantitative analysis of the tested drugs was also possible using color image analysis. A centrifugal microdevice was developed by Krauss et al. to perform simultaneous reactions with cobalt thiocyanate and sodium nitroprusside/acetaldehyde (Scott and Simon tests, respectively) [12]. The interpretation of the images obtained with a smartphone allowed the selective identification of COC and MET in a single run. Similarly, Lockwood et al. developed a paper-based analytical device to perform 12 colorimetric tests. This approach was applied to differentiate four common illicit drugs (heroin, COC hydrochloride, crack COC and MET) from 64 distractor and cutting agents [21]. The reactions were carried out in parallel resulting in characteristic bar codes, which were compared with images of a library for positive identification. Very recently, we developed a bi-colorimetric polydimethylsiloxane (PDMS) based device with two entrapped reagents, 1,2-naphtquinone-4-sulphonate (NQS) and KMnO_4_, that allowed the discrimination between SCP and MET through the observation of color changes produced in both the solution and the solid device [22].

So far, the multiplex detection approach remains relatively unexplored, and research is necessary to adapt classical chemical spot tests to solid formats. The availability of reactions with improved discrimination power will play a key role in the successful implementation of this kind of device [6,7,12].

In this study, we describe a highly selective colorimetric assay for AMP based on its reaction with gold bromide, a reagent proposed for the identification of ketamine (KET) through the formation of a dark precipitate of colloidal gold [23]. Recently, we adapted the test to a solid format by collecting the precipitate formed onto nylon membranes [24]. The strict control of the reaction time was necessary for the identification of KET because many other substances were oxidized with gold bromide producing similar color changes, with AMP being an exception. This drug reacted with gold bromide giving yellow–orange suspensions. Taking advantage of the different behavior observed for AMP, in this work we developed a colorimetric test that can be used for the selective identification and quantification of this drug. Although paper is the most common support in colorimetric sensors, alkaline gold bromide is not stable in contact with cellulose. For this reason, nylon membranes were selected to isolate the product of the reaction and for the subsequent measurement of the absorbance in quantitative studies. According to the literature, nylon was successfully used in combination with a variety of reagents [25,26,27] and, unlike cellulose filters, nylon membranes exhibit good compatibility with the reagent [24].

## 2. Results

### 2.1. Study of the Reaction and Selectivity

First, the reaction between alkaline gold bromide and AMP was studied. The reaction was also tested for solutions of other drugs such as MET, EPH, COC and SCP, as well as other compounds that are often used as diluents or adulterants, more specifically paracetamol, caffeine and procaine. For this purpose, 200 µL of solutions of the tested compounds were introduced in Eppendorf tubes and mixed with 200 µL of a solution 0.2 M of NaOH and 200 µL of a solution of gold bromide (0.5%, *w/w*) [24].

**Figure 1:**
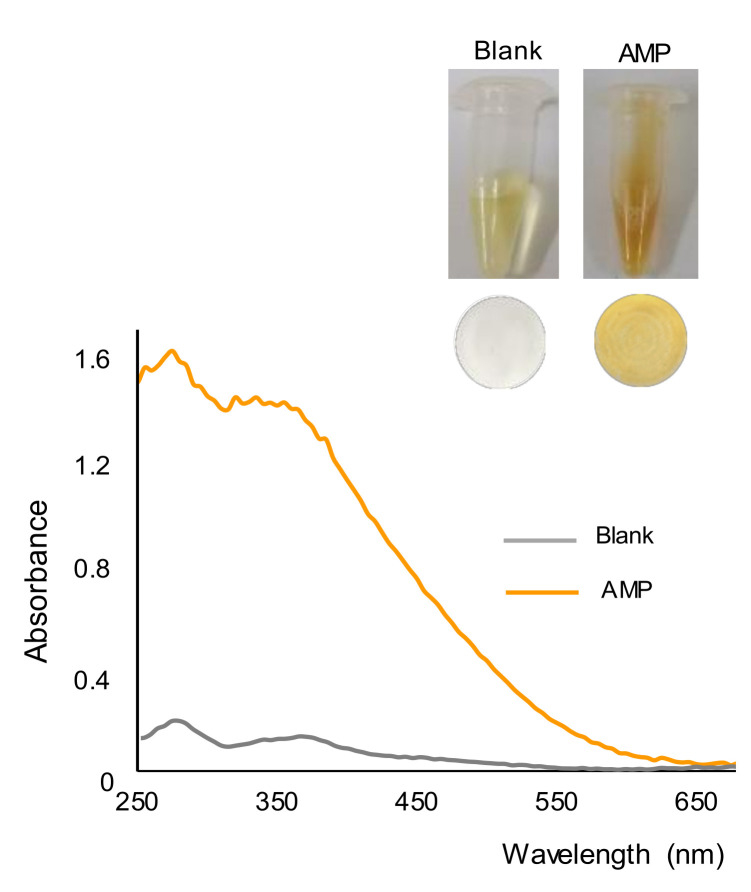
Pictures of the working solutions and membranes obtained for a blank (water) and a solution containing 500 ppm of AMP, and diffuse reflectance spectra of the membranes.

In the presence of AMP, a few seconds after the addition of the reagent, the formation of a yellow-orange precipitate was observed, which is in agreement with previous findings [24]. This can be seen in Figure 1, which shows images taken from the working solutions of a blank and AMP (500 ppm) after a reaction time of 15 min. This figure also shows the nylon membranes obtained after filtrating the working solutions or suspensions, as well as the reflectance diffuse spectra of the membranes. It was observed that the color of the membrane corresponding to the blank was rather similar to that of an unused membrane and clearly different from that obtained for the AMP solution. The positive identification of this drug with the membrane was easier than with the solution/suspension, as the color of the solutions of alkaline gold bromide was also yellow. The spectra of the two membranes were also different, with maxima differences of absorbance at 275 nm and 360 nm.

No significant changes were found for solutions of COC using reaction times up to 15 min. For the other drugs, the slow formation of a dark precipitate similar in appearance was observed, although for a given reaction time the amount of precipitate formed was significantly higher for EPH. A similar precipitate was observed for paracetamol. No changes were observed for solutions of caffeine, whereas the color of the solutions of procaine turned slowly to red.

As stated earlier, alkaline gold bromide was originally proposed by Sarwar as a reagent for the identification of KET [23]. The author reported that the formation of a dark precipitate of colloidal gold was indicative of the presence of KET, although the time of the reaction had to be strictly controlled because other substances were also reactive (particularly those with -OH groups) producing similar precipitates. The results found in the present study are consistent with those reported by Sarwar (EPH, SCP and paracetamol have -OH groups in their structure). Procaine, which was not included in Sarwar’s work, produced reddish solutions. The color observed for this compound (with no -OH groups in its structure) suggested the formation of gold nanoparticles. This was further confirmed by comparing the UV-vis spectra obtained for a solution of procaine treated with the reagent and a solution of gold nanoparticles (Supplementary Materials Figure S1). The exhaustive characterization of the products of reaction obtained for the potential interferents was considered beyond the scope of this study, considering that none of them interfere with the identification of AMP.

**Figure 2:**
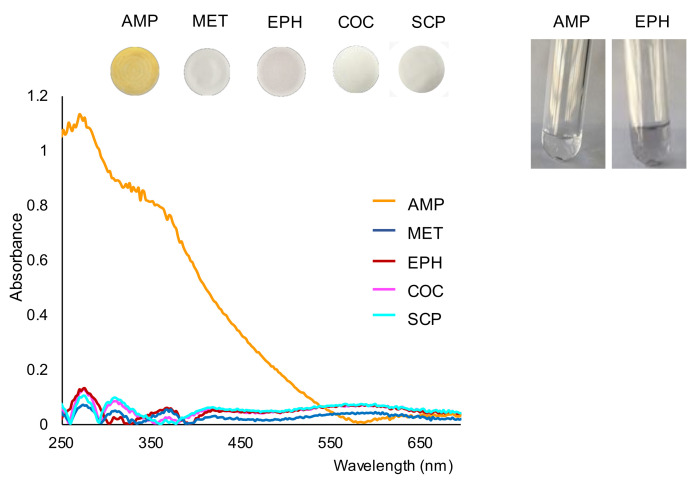
Images of the membranes obtained for the tested drugs and their respective reflectance spectra, and images of the working solutions of AMP and EPH taken 15 min after the filtration step. Concentration assayed, 500 ppm.

Next, the working solutions/suspensions were filtrated onto nylon membranes in order to separate any precipitate that originated. The images of the membranes obtained for the drugs tested are shown in Figure 2. As can be observed in this figure, the membrane obtained for AMP could be clearly differentiated from those obtained for the other drugs. This figure also shows the spectra recorded for the membranes. As can be seen, the spectra recorded for compounds other than AMP were quite similar and similar to those found for a blank (Figure 1).

**Figure 3:**
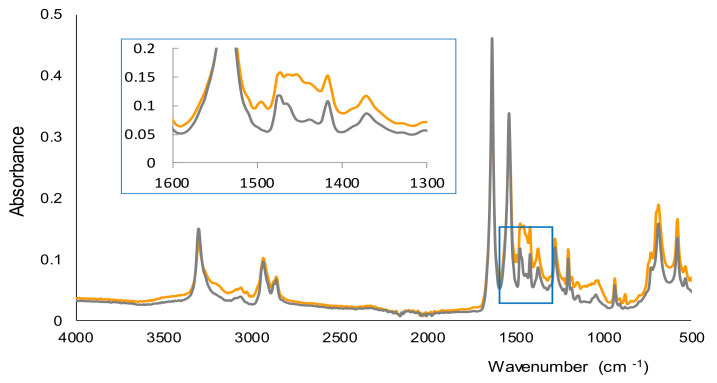
FTIR-ATR spectra of the membranes obtained for a blank (water) and a solution containing 500 ppm of AMP.

In Figure 3 the spectra recorded by FTIR-ATR for the membranes corresponding to a blank and a solution of AMP (500 ppm) are shown. Both spectra were quite similar, and according to a previous study, most of the bands observed were due to nylon and the water that remained in the membranes after the filtration stage [28]. The main differences between the two spectra of Figure 3 were found in the 1450–1500 cm^−1^ region. A band was observed in this region only for the membrane obtained for the solution of AMP, which can be attributed to the C=C stretching vibration of aromatic rings. This suggests that AMP, with an aromatic ring in its chemical structure, is part of the insoluble derivative collected onto the membranes. Nevertheless, the complete characterization of the derivative would require a more exhaustive study using other instrumental techniques.

Once filtered, the reaction solutions were collected into clean tubes and examined for possible changes. No additional formation of precipitate was observed in the solution collected for AMP, as can be observed in Figure 2. These results suggest that most part of the analyte had reacted before the filtration step. However, the formation of precipitate in the solution of EPH continued after the filtration stage (see also Figure 2). The formation of dark precipitate was also observed after filtering the SCP solution, although to a lesser extent. The above results indicate that the reaction between AMP and gold bromide was faster than the reaction with the other compounds tested. According to these observations, filtering the working solutions a short time after the addition of gold bromide would minimize the presence of colloidal gold in the membranes formed in the oxidation of EPE (or other compounds such as SCP, KET or paracetamol, if present). Nevertheless, the presence of EPH could be easily differentiated from the AMP-derivative by either the color of the membranes or by the reflectance diffuse spectra of the membranes.

### 2.2. Quantitative Performance

**Figure 4:**
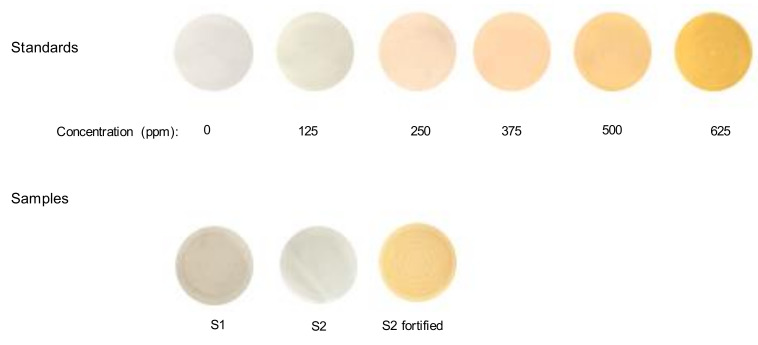
Images of the membranes obtained for the calibration solutions and for the drug-street samples.

Next, the quantitative performance of the method was studied. As stated earlier, the reaction between AMP and gold bromide was very rapid, and the yellow–orange precipitate of the derivative formed could be observed a few seconds after the addition of the reagent. Nevertheless, for better control of the amount of precipitate collected, after a reaction time of 2 min, the resulting suspensions were filtered. Solutions of AMP at concentrations ranging from 125 to 1000 ppm were tested under the proposed conditions, and then the absorbance of the collected membranes was measured at 275 nm. A linear variation of the analytical signal was observed up to concentrations of the drug of 625 ppm. Figure 4 shows the images of the membranes obtained for solutions of the drug within the linear working interval. It has to be noted that the minimum amount of AMP detectable visually was 250 ppm.

The calibration equation was calculated by plotting the absorbances measured for the standards (after subtracting the absorbance measured at the working wavelength for the membrane corresponding to a blank) against the concentration of AMP. Table 1 lists the calibration parameters obtained.

The precision was evaluated by processing three replicates of standard solutions containing 250 and 500 ppm of AMP, respectively. The RDS values are also listed in Table 1.

Next, the proposed method was applied to the analysis of binary mixtures of AMP and other compounds. EPH and caffeine were selected as the model of drug and diluent, respectively; they can also be considered representative of compounds reactive and non-reactive towards gold bromide. The compositions of the mixtures assayed are given in Table 2. Portions of the mixtures were diluted with the appropriate volumes of water in order to produce final concentrations of AMP of 400 ppm. Then, the solutions were processed under the proposed conditions. The percentages of AMP in the mixtures were calculated from the absorbance values measured for the membranes and the calibration equation of Table 1. The results found are summarized in Table 2. As observed, the values obtained were close to the true values, even for the sample containing an EPH to AMP percentage ratio of three.

### 2.3. Digital Color Analysis of the Filters

The possibility of using the filters for the quantitative analysis of AMP by digital color analysis was also evaluated. The numerical red–green–blue (RGB) and cyan–magenta–yellow-key (CYMK) color systems were tested. The color coordinates were then measured using the central area of the filters. The coordinates obtained for the blank were subtracted from the values measured for the standard solutions. The best results were obtained with the blue coordinate, which provided suitable linearity and precision, as can be seen in Table 1.

### 2.4. Application to the Analysis of Drug Street Samples

The proposed method was applied to two different drug street samples. According to the results obtained by liquid chromatography, one of the samples (S1) contained KET, and the other (S2) was an AMP-type sample with MET and 3,4-metthylenedioxymethamphetamine (MDMA) [16,17]. Portions of 0.4 g of the samples were treated with 1 mL of water and filtered, and then processed by the proposed procedure. The images of the membranes obtained for the two samples are shown in Figure 4. As observed, none of them gave the yellow–orange membranes characteristic of AMP, which is consistent with the results obtained by the reference technique [16,17].

Finally, a portion of the solution obtained for the AMP-type drug street sample was fortified with AMP to a final concentration of 400 ppm, and the solution was treated by the proposed method. Figure 4 shows an image of the membrane collected. The absorbance of the membrane was used to calculate the concentration of AMP in the sample, using the calibration equation of Table 1. The results are listed in Table 3. As can be deduced, the concentration of AMP found in the sample was statistically equivalent to the concentration added (*t_calculated_* = 0.866, *t_critical/95%_* = 4.303). For comparative purposes, the filters were also analyzed by the color image analysis approach. As can be seen in Table 3, the results were statistically comparable to those obtained by diffuse reflectance (*t_calculated_* = 1.010, *t_critical/95%_* = 2.776).

## 3. Discussion

Today, the rapid identification of drugs is in demand in areas such as point-of-care assistance, prosecution of drug trafficking or saving resources in laboratories by applying previous screening tests (only positive samples undergo further analysis by sophisticated and time-consuming instrumental methods). The application of portable instruments such as infrared [29] and surface-enhanced Raman spectroscopy [30] and liquid chromatography [22,31] for the analysis of drugs is in constant growth. However, because of their simplicity, speed and low cost, chemical spot tests are still widely used in a variety of contexts. Moreover, the combination of classical chemical spot tests with recent advances in fields such as the development of microfluidic devices, solid (bio)sensors and multiplexed analysis has opened new possibilities. Another advantage is that colorimetric assays can be easily adapted to quantitative analysis with a minimum of extra work. However, the main drawback of colorimetric assays, which is their poor selectivity, persists. In addition, many of the classical reactions proposed for the detection of drugs involve the use of corrosive reagents, which limit their implementation in on-site tests [6]. Therefore, the development of new colorimetric assays that enhance selectivity while reducing the hazards for the operators is of great interest.

In this study, a new colorimetric assay is proposed for AMP based on its reaction with alkaline gold bromide. This reagent is reactive towards different drugs and compounds commonly found in illicit drug street samples, giving different forms of elemental gold. However, we demonstrated here that the reaction with AMP leads to the formation of an insoluble AMP-derivative which can be easily distinguished from the responses of other common drugs. Although the formation of an insoluble yellow–orange derivative is a first indication of the presence of AMP, the product of the reaction can be collected onto a nylon membrane, which facilitates the identification (the solutions of reagent are also yellowish) and the quantification of the drug, if necessary.

**Figure 5:**
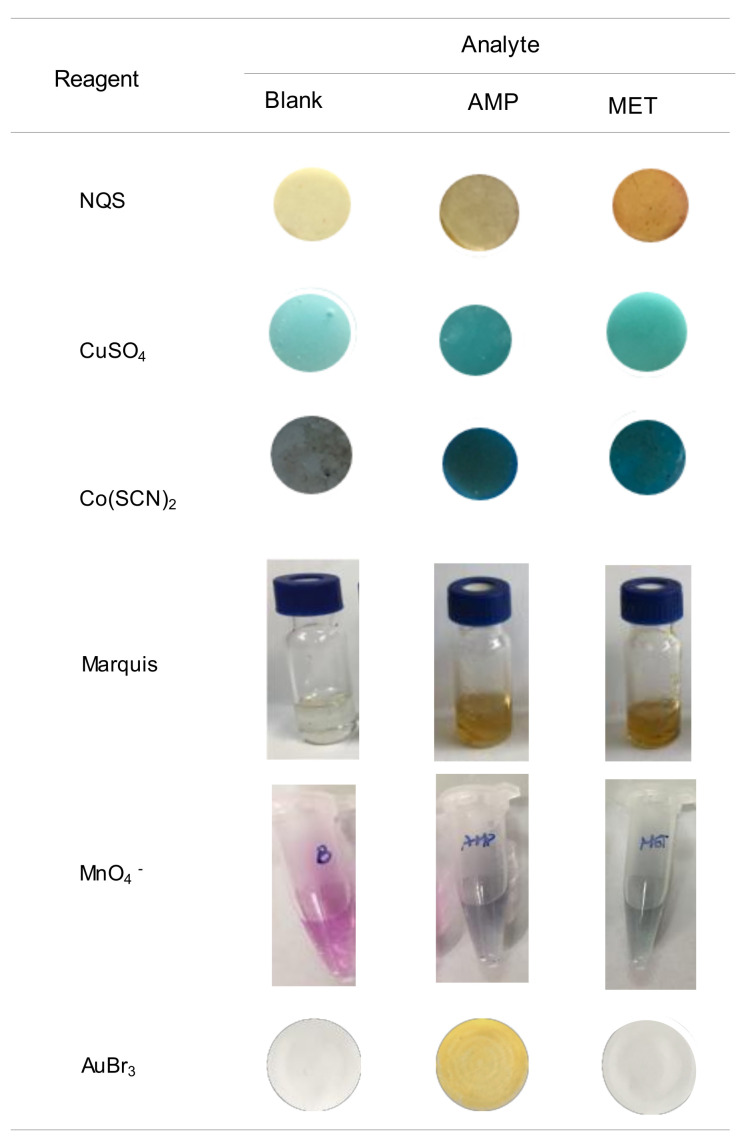
Images obtained for a blank and solutions of AMP and MET with different PDMS-sensors previously proposed for the analysis of drugs, and with the proposed assay.

The high selectivity towards AMP makes this assay very attractive, in particular for the differentiation between AMP and MET. These two compounds can barely be differentiated by other common tests. In Figure 5 the images obtained for these two compounds with some tests developed for drugs using PDMS-based sensors [16,17,18,19,24] are compared. In all instances, the reagents were entrapped into the polymeric PDMS network, although the response was generated by different mechanisms. When exposing the NQS, CuSO_4_ and Cu(SCN)_2_ sensors under alkaline conditions to the sample solutions, the analytes diffuse from the solutions to the PDMS network causing a change of color of the PDMS sensor. For the Marquis test and the test with MnO_4−_, the reagent diffuses from the sensor to the working solution; a change of color of the working solution is indicative of the presence of the suspected drug.

As observed from this figure, solutions of AMP and MET led to similar colors by the Marquis tests, which is probably the most employed test for the screening of unknown drug street samples [6]. The two compounds are reactive towards Cu(SCN)_2_, which can be used under different conditions for the identification of COC and KET [17], and towards CuSO_4_ a reagent proposed for the identification of EPH and derivatives (Chen–Kao test) [19]. Only the NQS sensors allowed the differentiation of the two compounds; this reagent forms derivatives with different colors depending on whether they contain primary (brown) or secondary (yellow) amino groups [16].

As regards, the sensor of KMnO_4_, developed for SCP, both AMP and MET reacted producing a change of color of the working solutions from violet due to the MnO_4_^−^ ion to green (MnO_4_^2−^), although the reaction with MET was faster. This means that the differentiation between the two compounds would require strict control of the reaction time, taking into account that in all instances the reaction led to the same product of the reaction. Indeed, SCP would be an interferent, as well as other compounds that can be easily oxidized. Similar results were also reported when using other common spot tests that involve oxidizer agents such as molybdic acid or MnO_4_^−^ (Frohede test) [6,11]. A modification of the Simon test, which is commonly used for the presumptive analysis of drugs containing primary and secondary amino groups, was developed for a more selective identification of AMP [13]; the conventional and modified reactions had to be applied to differentiate AMP from MET.

As can be derived from Figure 5, the main advantage of the proposed reaction is that AMP can be easily differentiated from MET. In addition, unlike most of the colorimetric (bio)assays developed for drugs, which may involve sophisticated and time-consuming synthesis of the recognition probes [6,7,20], the reagent is commercially available. The reaction is very rapid and does not involve the employment of strong acidic media, so it is well-suited for on-site tests. As regards the accuracy, the relative errors found in the analysis of a fortified street sample ranged from −4% to +12% (see Table 3). The analysis of different amphetamines using PDMS sensors with entrapped formaldehyde (Marquis) and CuSO_4_ reagents ranged from +0.3% to +14% and −1.3% to +10%, respectively [18,19]. Values ranging from 0% to +20% were found with other colorimetric methods developed for AMP [13]. Therefore, it can be concluded that the proposed method provides accuracy comparable to that achieved with other methods developed for the rapid analysis of drugs.

The reaction can be used under a conventional chemical spot-test format (using blister packs or glass ampoules, for example): the formation of a yellow–orange precipitate would indicate a positive response for AMP. However, a simple filtration can be carried out to collect the precipitate (filtration takes less than 1 min). In such a way, the membranes make the response less subjective (solutions of gold bromine are yellow), and for samples that test positive they can be further processed to obtain quantitative information. For this purpose, reflectance diffuse measurements can be used. Good quantitative performance was found within the AMP concentration interval 125–600 ppm, although the minimum concentration of AMP detectable visually was 250 ppm. The quantitative performance is similar to that reported in other colorimetric tests based on solid sensors in terms of precision and sensitivity [16,17,18,19]. The method provides adequate results even in the presence of an excess of EPH, a compound that can be oxidized by gold (III). Satisfactory results were also obtained for the analysis of illicit drug-street samples. The proposed membrane-based sensor is compatible with color analysis images, which would facilitate its application for on-site analysis.

Another important advantage is that, because of its solid format, the tests could be integrated into multiplexed approaches such as those involving microfluidic devices. The fact that the reaction does not involve strong acidic media, which may be incompatible with supports used in this kind of device [11], is a good starting point. Alternatively, the reagent could be entrapped in a polymer and then combined with other reagents to form solid sensors with enhanced detection capabilities, for example by using a design similar to the PDMS bi-colorimetric sensor developed in [22]. Since gold bromide is not stable in PDMS [24] future research will be necessary to find compatible materials that can be used in the design of this kind of device.

## 4. Materials and Methods

### 4.1. Reagents and Solutions

All the reagents were of analytical grade and purity ≥ 97%. Gold bromide, COC hydrochloride, AMP sulfate, MET hydrochloride, EPH hydrochloride, SCP hydrobromide and procaine were obtained from Sigma-Aldrich (St. Louis, MO, USA). Sodium hydroxide was supplied by Panreac (Barcelona, Spain). Paracetamol and caffeine were purchased from Guinama (Alboraya, Spain).

Stock solutions of the compounds assayed at a concentration of 1000 ppm were prepared by dissolving the pure reagents in water. The stock solutions were stored in the dark at 4 °C until use. Working solutions were prepared by diluting the stock solutions with ultrapure water. Ultrapure water was obtained from an Adrona system (Riga, Latvia).

### 4.2. Reaction Conditions

A solution containing 0.5% of gold bromide (*w/w*) in ultrapure water was used as derivatization reagent. Aliquots of 200 µL of the working solutions were mixed 200 µL of a solution of 0.2 M NaOH in 1.5 mL Eppendorf tubes. Then, 200 µL of the gold bromide solution was added. Unless otherwise stated, the reaction time was 2 min. Next, the resulting suspensions were aspirated with 1 mL-polypropylene syringes, and then filtered through nylon filter membranes of 13 mm of diameter and 0.45 µm of pore diameter (GE Healthcare, Buckinghamshire, UK); the membranes were placed inside a 13 mm-polypropylene filter holder (Millipore Corporation, Billerica, MA, USA). All assays were carried out in triplicate.

In the comparative study of the responses obtained with other colorimetric sensors (Figure 5), the working conditions were as follows. For the sensors with NQS, CuSO_4_ and Co(SCN)_2_, aliquots of 200 µL of the target compounds and 200 µL of 0.1 M NaOH were introduced into vials; then, the sensors were immersed into the resulting mixture. After 30 min of exposure, the sensors were removed and photographed. For the modified Marquis test, 500 µL of concentrated sulfuric acid were mixed with 30 µL of solutions containing 1000 ppm of the analytes (final concentration in the solution, 60 ppm). Then, the sensors were introduced into the mixtures. The pictures of Figure 5 were taken 5 min after the introduction of the sensors into the working solutions. In assays with the KMnO_4_ sensors, aliquots of 600 µL of the standard solutions of the analytes (500 ppm) prepared in a mixture of acetonitrile: 0.1 M NaOH (1:2, *v/v*) were exposed to the sensors for 20 min. Then, the sensors were removed and the pictures of the working solutions were taken.

### 4.3. Instrumentation and Conditions

Spectrophotometric measurements of the nylon membranes with the product of reaction obtained with gold bromide were carried out using a Cary 60 Fiber Optic UV-Vis spectrophotometer (Agilent Technologies, Waldbronn, Germany), fitted with a remote fiber optic diffuse reflectance accessory purchased from Harrick Scientific Products (Mulgrave, Victoria, Australia). Data were recorded and processed using Cary WinUV software (Agilent Technologies, Waldbronn, Germany). The spectra were recorded in the 250–700 nm range in diffuse reflectance mode. The working wavelength selected for quantitative studies was 275 nm.

Photographic images of the nylon membranes were taken with the camera of smartphones at an approximate distance of 20 cm. The images were then imported into a computer, and color analysis was carried out with the free image editor software GIMP (version 2.8.18). The color intensity was analyzed using the color picker tool of GIMP with an average of 35 × 35 pixels in the center of the membranes. The numerical RGB and CYMK color systems were used to transform the images into numerical coordinates.

Infrared spectra measurements were carried out with a Cary 630 FTIR-ATR spectrophotometer Agilent Technologies (Böblingen, Germany). Spectra were recorded in the frequency range of 4000–600 cm^−1^ at a resolution of 4 cm^−1^. For data collection and processing, MicroLab FTIR and ResolutionPro software (Agilent Technologies, Waldbronn, Germany) were used, respectively.

### 4.4. Application to the Analysis of Drug Street Samples

The real drug street samples used in this study were voluntarily donated by an individual who was previously informed of the aim of the study. According to previous results, one of the samples was positive for KET (S1), while the other (S2) was an AMP-like drug-street sample that contained MET and MDMA [16,17]. Accurately weighed portions of the powdered samples were dissolved in the appropriate volume of nanopure water, so that the resulting solutions contained 0.4 g of sample per mL. Then the samples were filtered through 0.22 μm nylon membranes supplied by GVS (Sanford, ME, USA). Finally, aliquots of 200 μL of the resulting solutions were transferred and processed by the proposed method.

## 5. Conclusions

A new colorimetric test is proposed for the rapid identification and quantification of AMP in drug street samples. The assay is based on the employment of gold bromide, a readily available reagent, and subsequent filtration of the insoluble AMP derivative originated. The main advantage of the proposed procedure is that it is highly selective for AMP, thus AMP can be easily differentiated from other common drugs such as MET, COC, SPC or EPH and potential diluents (caffeine, paracetamol, procaine). Moreover, the filters can then be further processed for the quantitative analysis of this drug by reflectance diffuse spectroscopy (or by color image analysis). The method was successfully applied to the analysis of synthetic binary mixtures and illicit dug street samples.

Because of its solid format, the proposed approach could be an option to be considered in the development of multiplexed detection systems, a strategy that is becoming increasingly popular in drug analysis.

## Figures and Tables

**Table 1:** Calibration equations and precision obtained for AMP with the proposed method.

Method	Linearity, y = ax + b (*n* = 15)			Precision, RDS (*n* = 3) (%)
	a ± S_a_	b ± S_b_	R^2^	
Absorbance	0.0011 ± 0.0001	−0.14 ± 0.01	0.990	9 ^1^
				8 ^2^
Color Image Analysis	0.19 ± 0.02	0.42 ± 0.08	0.993	3 ^1^
				4 ^2^

^1^ For a concentration of 250 ppm; ^2^ for a concentration of 500 ppm.

**Table 2:** Results obtained for binary mixtures with the proposed method (*n* = 3).

Sample	Percentage of AMP in the Sample (%)	Percentage of AMP Found (%)	E_r_ (%)
AMP + EPH	25	24 ± 4	−4
	75	76 ± 8	+1
AMP + Caffeine	25	26 ± 2	+4
	75	77 ± 4	+3

**Table 3:** Results obtained for sample S2 fortified with 400 ppm of AMP (*n* = 3).

Analytical Signal	Found Concentration, (*n* = 3) (ppm)	E_r_ (%)
Absorbance	420 ± 40	−4
Color Coordinate (Blue)	448 ± 18	+12

## Data Availability

The data presented in this study are available from the authors.

## Supplementary Materials

The following are available online. Figure S1: Images and UV/vis spectra obtained for solutions of procaine treated with alkaline gold bromine and UV/spectrum of a solution of gold nanoparticles.

## Sample Availability

Samples of the compounds are available from the authors.
