# Supplementary material for: A Colorimetric Membrane-Based Sensor with Improved Selectivity towards Amphetamine
Source: Molecules. 2021 Nov 5;26(21):6713. doi: 10.3390/molecules26216713 (PMC8588055; doi:10.3390/molecules26216713)
Supplement: Supplementary file 1 [file molecules-26-06713-s001.zip › molecules-1421280-supplementary.pdf]

molecules

# A Colorimetric Membrane-Based Sensor with Improved Selectivity Towards Amphetamine – Supplementary Matterials

**Neus Jornet-Martínez, Pilar Campíns-Falcó and Rosa Herráez-Hernández ***

MINTOTA Research Group, Departament de Química Analítica, Universitat de València, Dr. Moliner 50, 46100-Burjassot, València, Spain, Neus.Jornet@uv.es (N.J.-M.); pilar.campins@uv.es (P.C.-F.)

* Correspondence: rosa.herraez@uv.es; Tel.: + 34-96-3544978

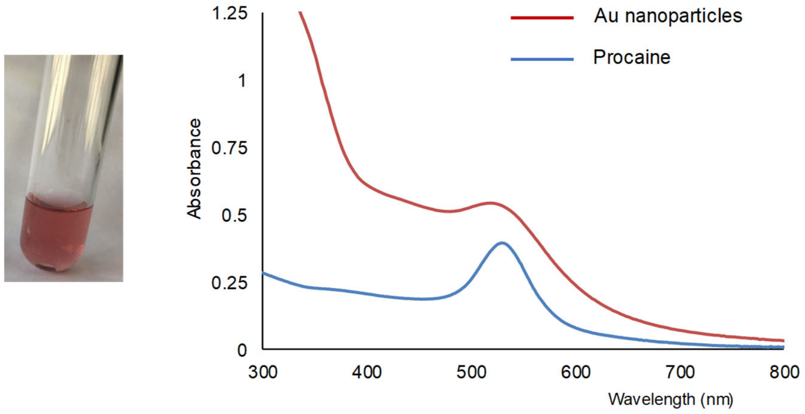


**Figure S1.** Images and UV/vis spectra obtained for solutions of procaine treated with alkaline gold bromine and UV/spectrum of a solution of gold nanoparticles.
